# Cutaneous reactive angiomatosis: a case report of a reactive manifestation of underlying acute myelogenous leukaemia

**DOI:** 10.1093/skinhd/vzag097

**Published:** 2026-07-03

**Authors:** Prajwal Pudasaini, Sushil Paudel, Sagar GC, Samir Neupane, Esna Thapa, Sadiksha Adhikari, Laxman Chapagain

**Affiliations:** Department of Dermatology, Civil Service Hospital, Kathmandu, Nepal; Department of Dermatology, Civil Service Hospital, Kathmandu, Nepal; Department of Dermatology, Civil Service Hospital, Kathmandu, Nepal; Department of Pathology, Civil Service Hospital, Kathmandu, Nepal; Department of Medicine, Sahid Gangalal National Heart Centre, Kathmandu, Nepal; Deparment of Dermatology, Gandaki Medical College Teaching Hospital, Pokhara, Nepal; Department of Dermatology, Tribhuvan University Teaching Hospital, Kathmandu, Nepal

## Abstract

Cutaneous reactive angiomatosis (CRA) is a rare entity which is a manifestation of an underlying localized and/or systemic condition. Notably, this condition is a reactive phenomenon with clinical manifestation and a tell-tale sign of underlying infective, autoimmune and lymphoproliferative conditions.

Dear Editor, A 35-year-old woman presented with painful erythematous plaque with a central vesicle studded on the surface of the inner aspect of her forearm for 3 days (Figure 1). The plaque ­progressed with the formation of vesicles on the surface that ­coalesced to acquire a size of 6 × 5 cm, with oozing alongside ­yellowish encrustation (Figure 2). The patient had a fever during onset of the lesion with a maximum temperature of 40 °C, along with malaise and myalgia. On total body skin examination, an indurated encrusted plaque with erosion and exfoliation were present over other plaques on the extensor aspect of the right lower leg (Figure 3). There was also a healed scar from a prior similar lesion that had erupted 6 months earlier, with similar progression over the upper back on the lateral aspect. A biopsy was taken from the forearm lesion, which showed scattered proliferation of round-to-elongated capillaries in the superficial dermis. The ­capillaries had prominent endothelial cells with fibrosis of the surrounding dermis (Figures 4–8). A routine blood investigation was carried out, along with a peripheral blood smear, which showed numerous blast cells suggestive of acute leukaemia. A bone marrow biopsy along with a cytogenetic test was done to confirm it, eventually, as a case of acute myelogenous leukaemia (AML).

**Figure 1:**
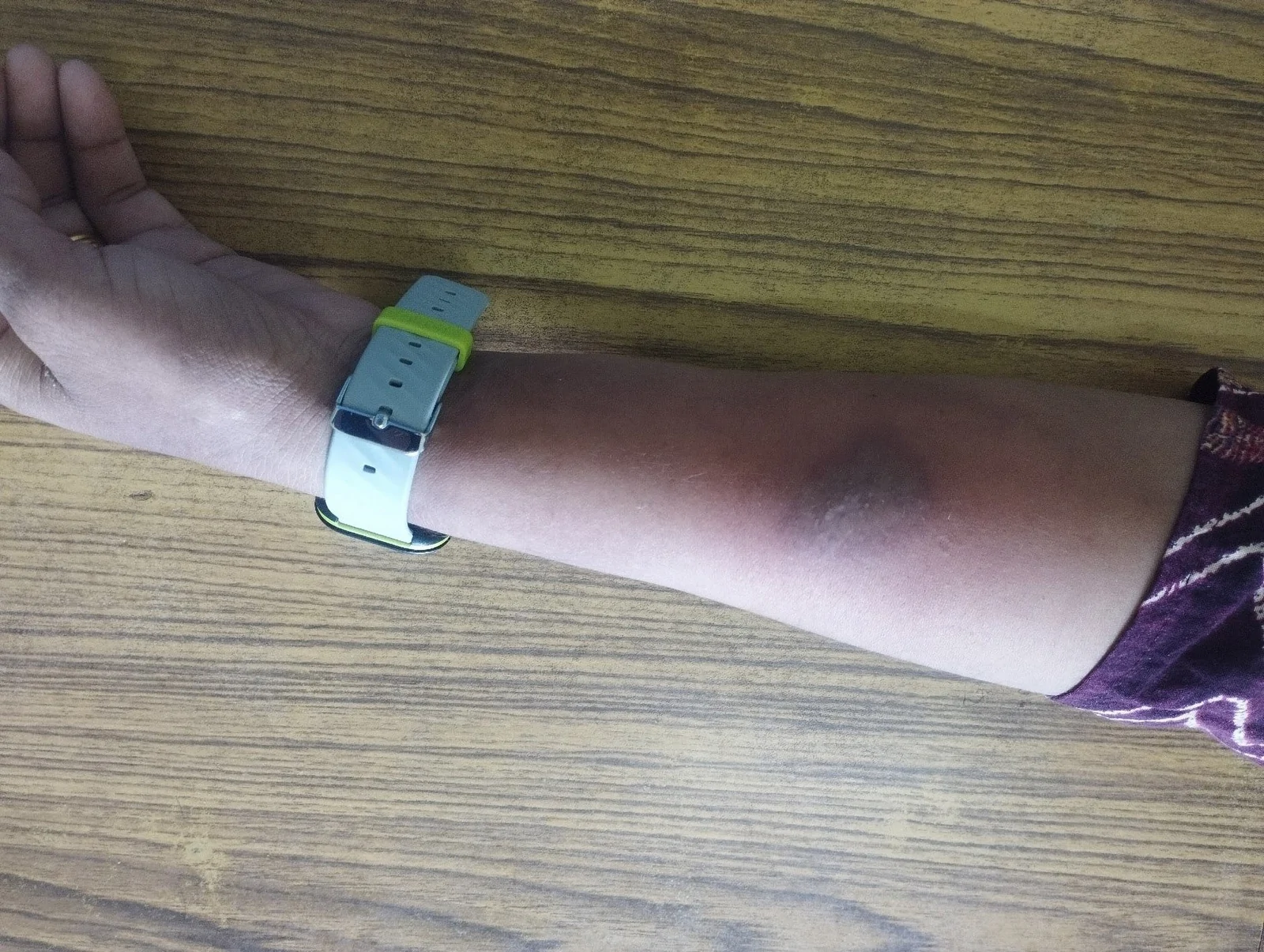
Painful erythematous plaque with a central vesicle studded on the surface over the inner aspect of forearm.

**Figure 2:**
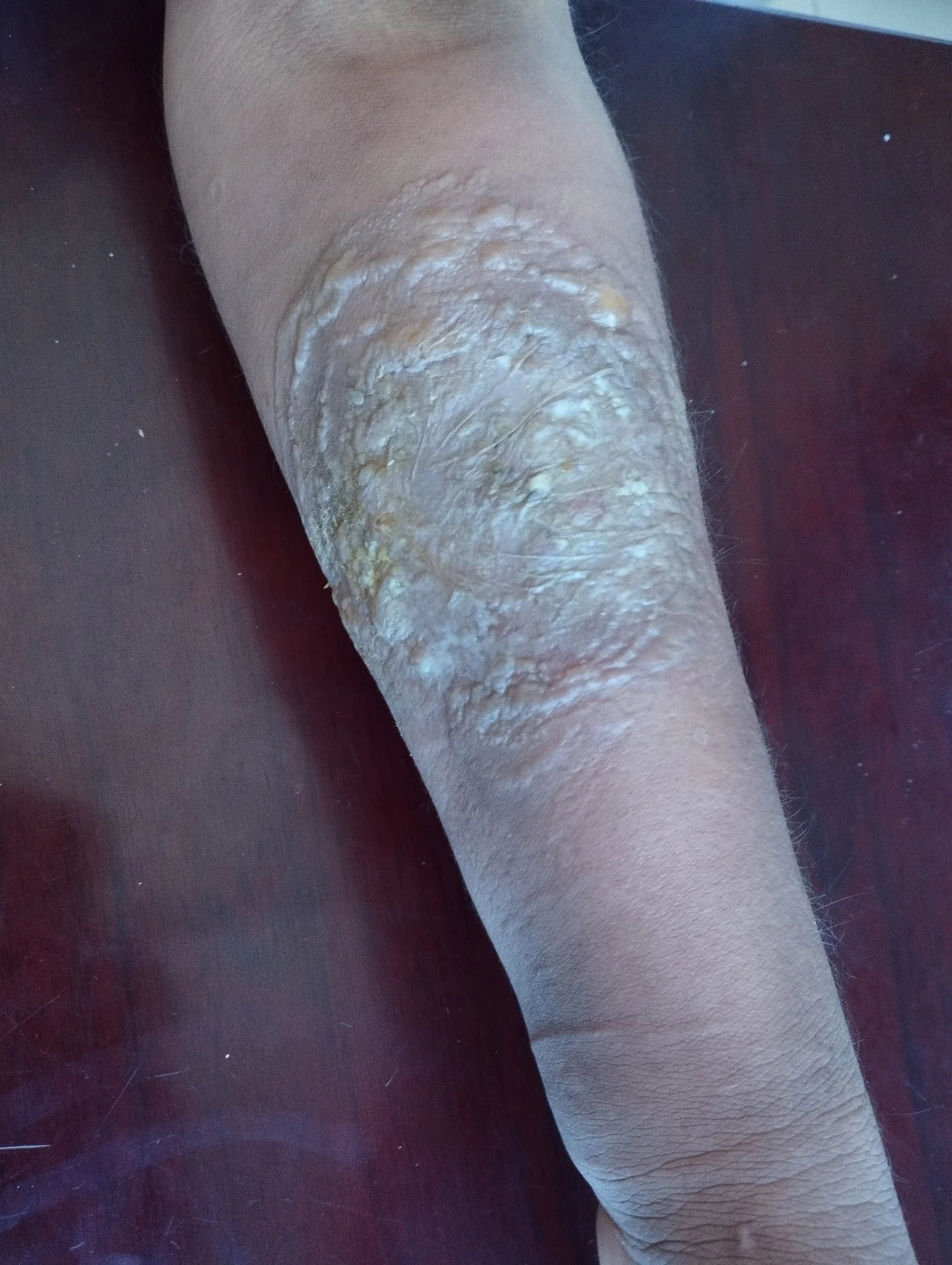
Plaque progression with the formation of vesicles on the surface, which coalesced to acquire a size of 6 × 5 cm, with oozing alongside yellowish encrustation over the volar aspect of the forearm.

**Figure 3:**
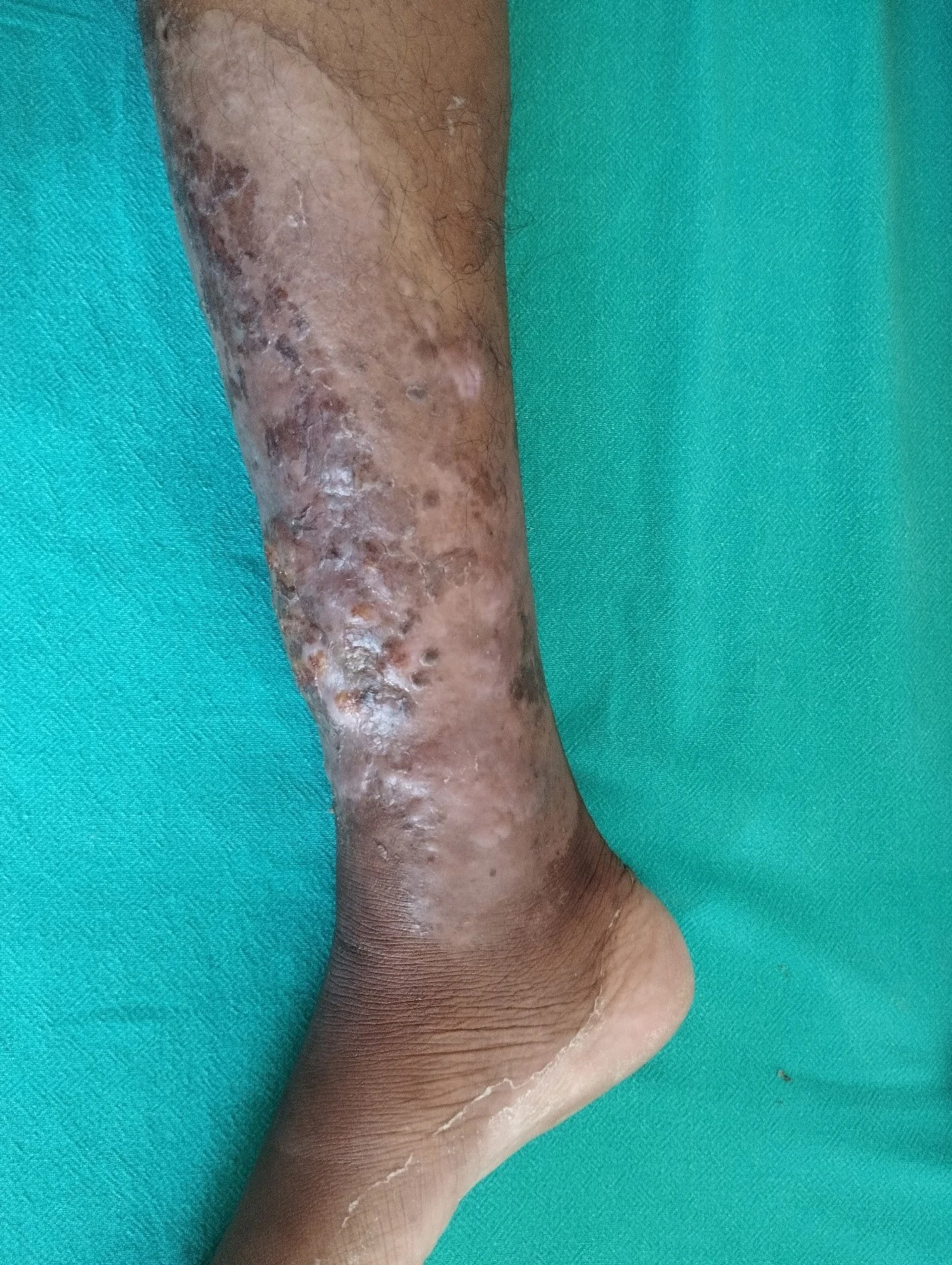
Indurated encrusted plaque with erosion and exfoliation present over another plaque on the extensor aspect of the right lower leg.

**Figure 4:**
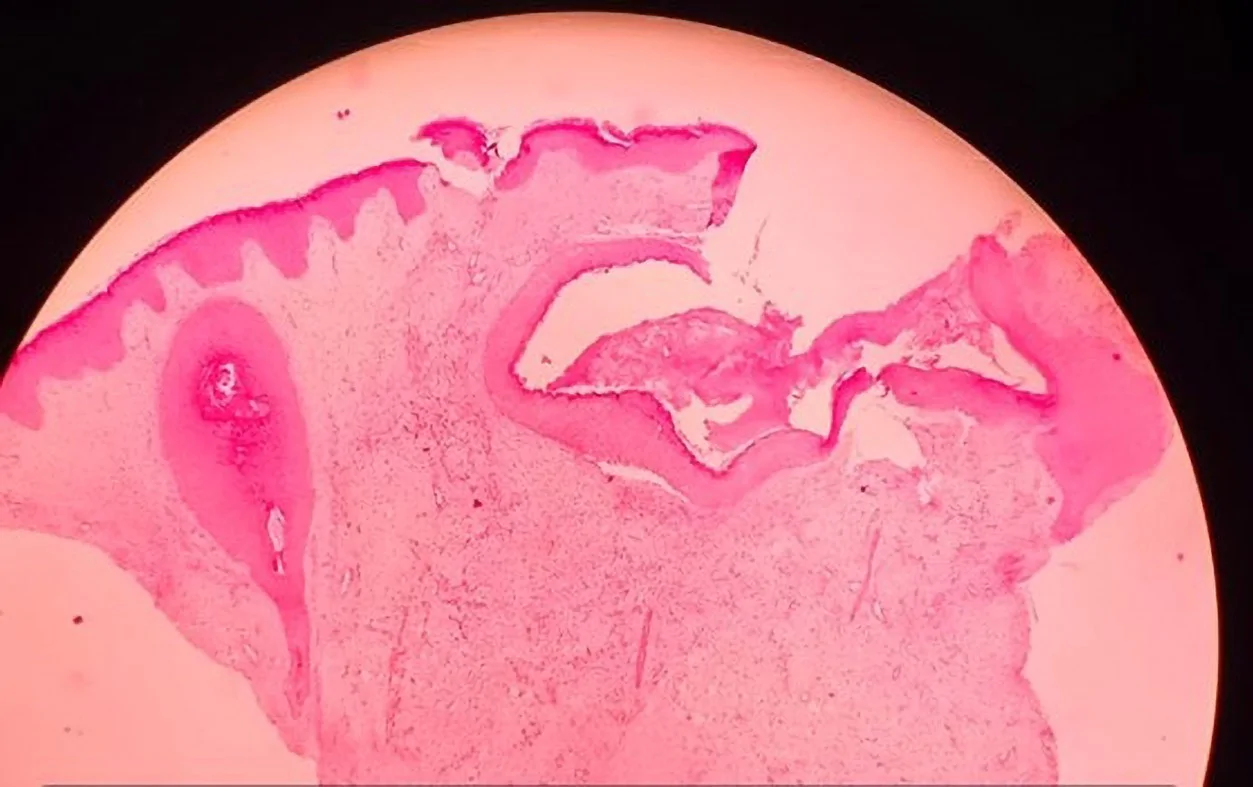
Haematoxylin and eosin staining (low-power view, ×4 magnification) of the biopsy specimen of the forearm skin lesion showing scattered proliferation of round-to-elongated capillaries in the superficial dermis.

**Figure 5:**
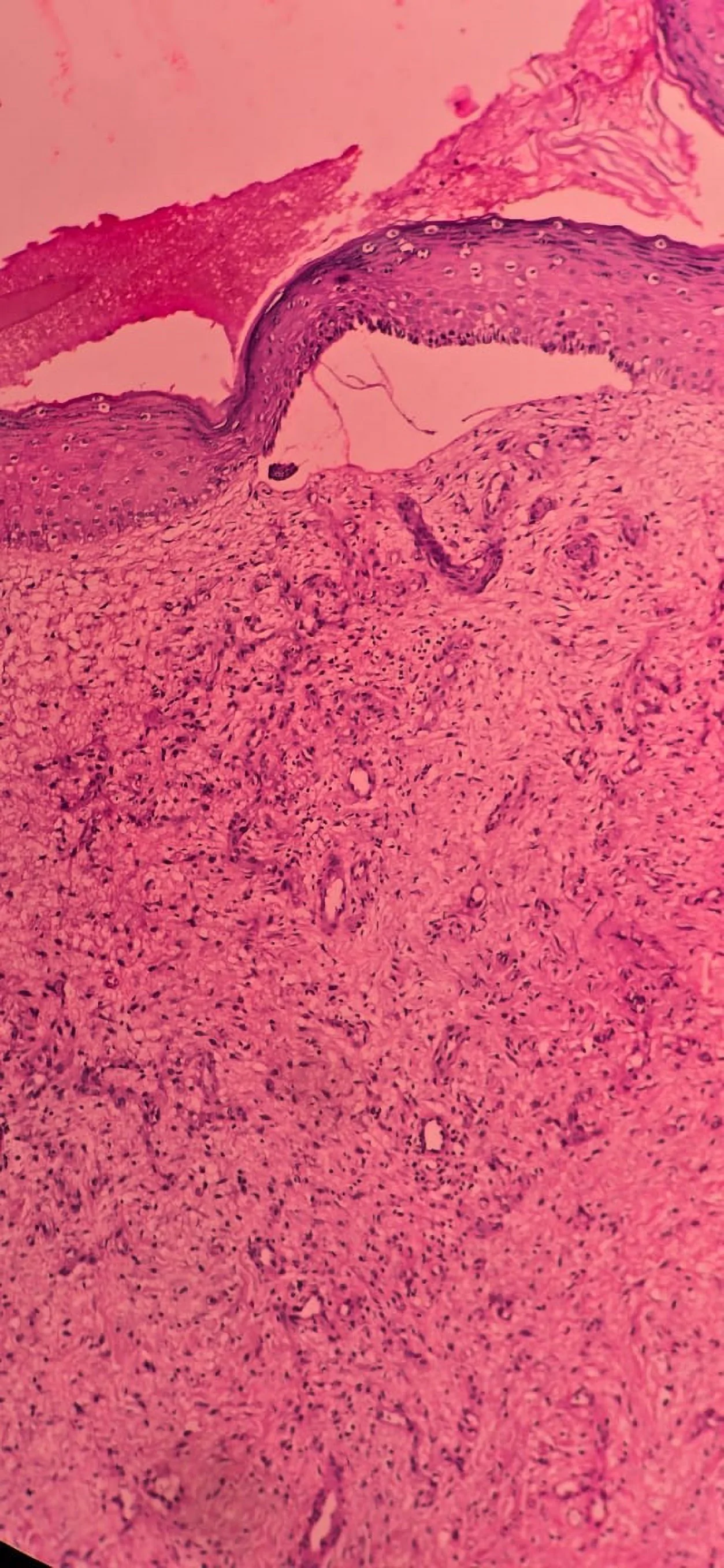
Haematoxylin and eosin staining (low-power view, ×10 magnification): section shows acanthosis and mild hyperkeratosis. Dermis shows proliferation of scattered capillary sieve-like round-to-elongated vessels in the dermis, which are prominent in the superficial dermis. The capillaries show proliferation of endothelial cells, along with aggregates.

**Figure 6:**
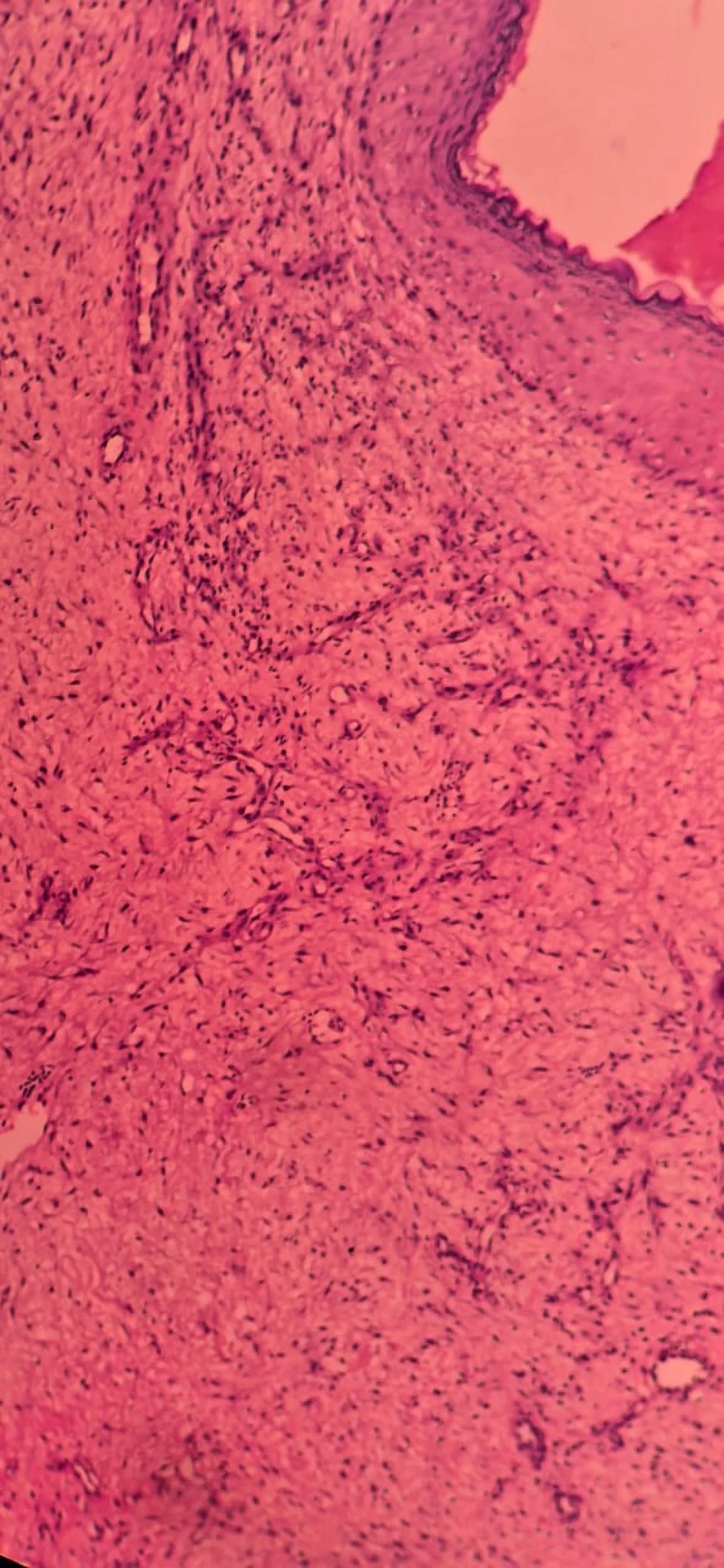
Haematoxylin and eosin staining (low-power view, ×10 magnification): dermal section shows the proliferation of scattered capillary sieve-like round-to-elongated vessels in the dermis, which are prominent in the superficial dermis. The capillaries show proliferation of endothelial cells, along with aggregates. The dermis also shows fibrosis.

**Figure 7:**
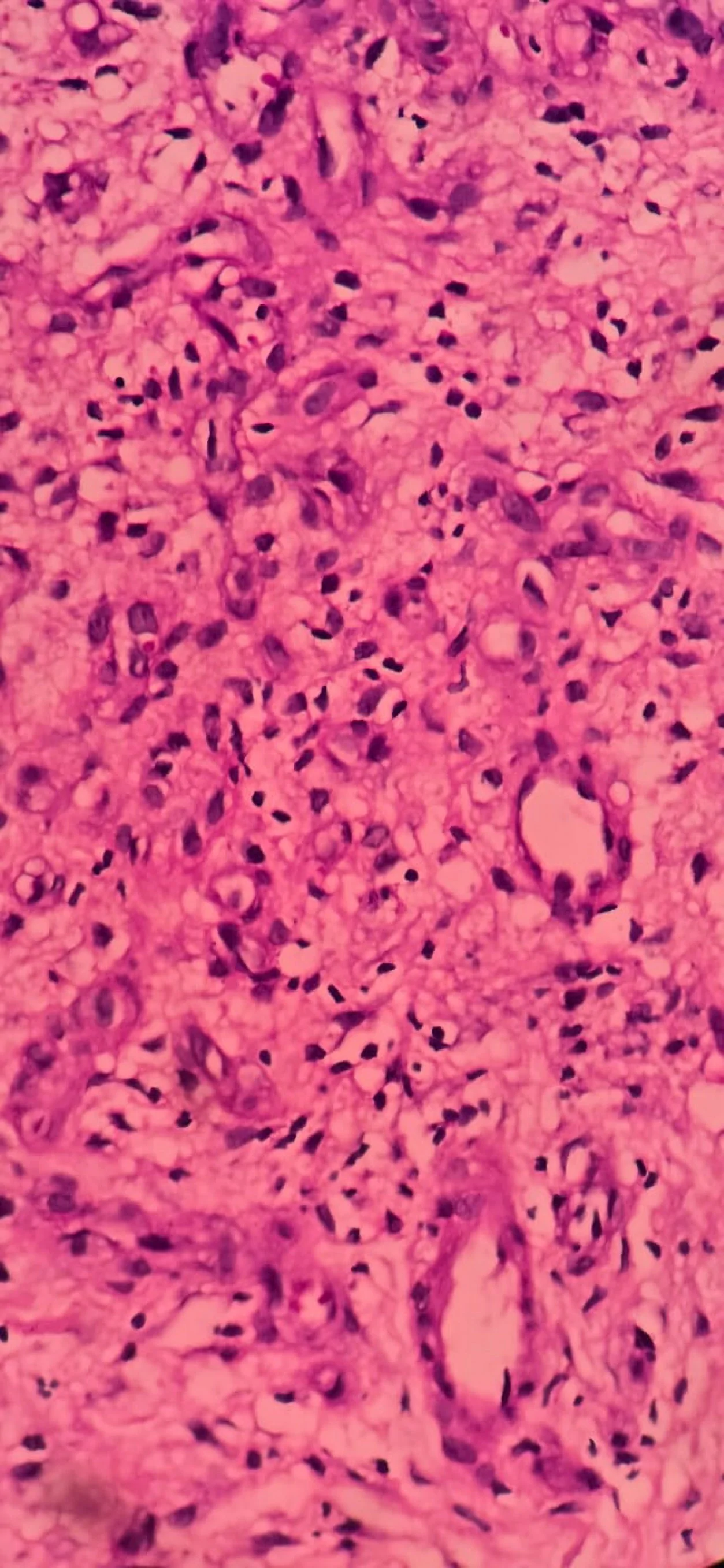
Haematoxylin and eosin staining (×40 magnification): perivascular spindle cells suggestive of pericytes are seen surrounding capillary-sized vessels. No cellular atypia is seen.

**Figure 8:**
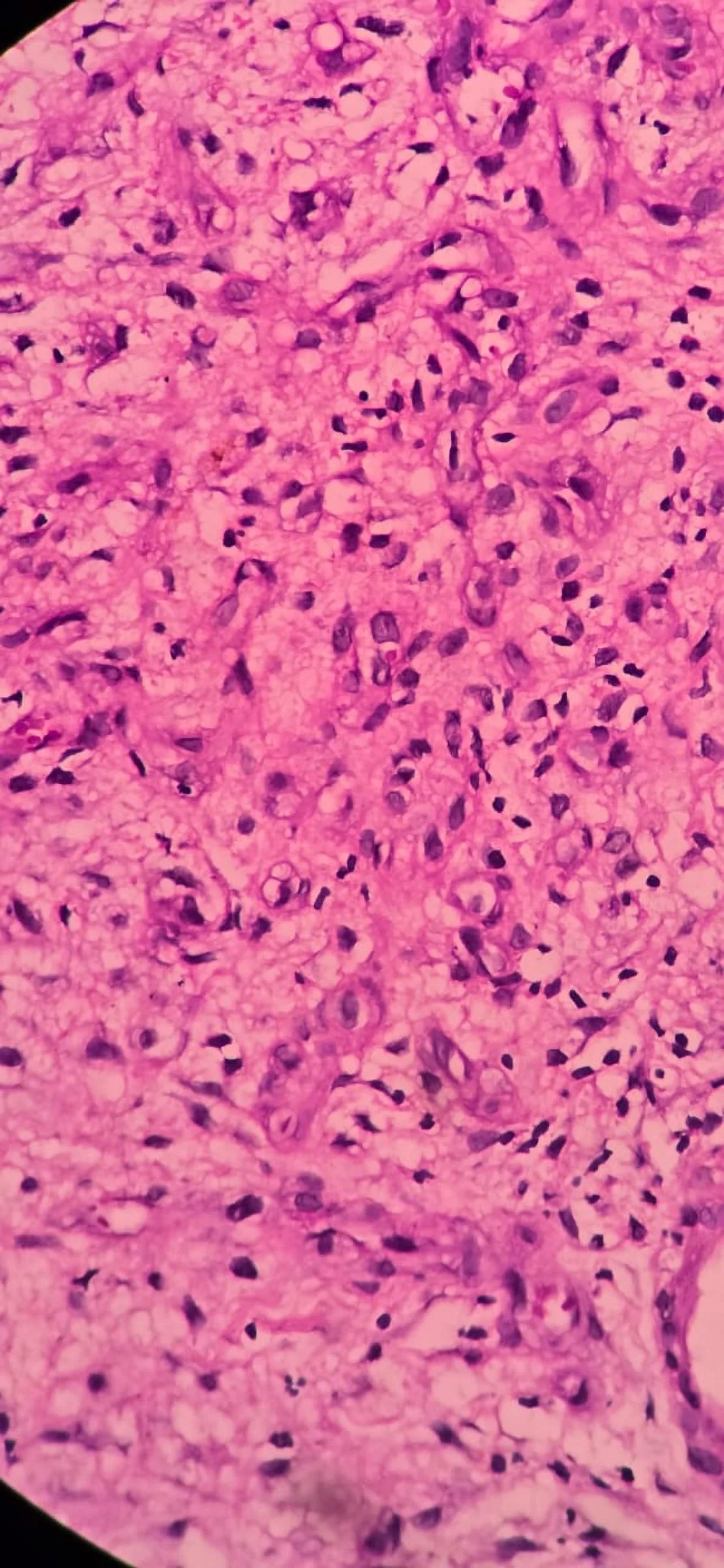
Haematoxylin and eosin stain (×40 magnification): section shows clear visualization of individual endothelial cell nuclei, perivascular spindle cells, fine stromal details and fibrosis.

Cutaneous reactive angiomatosis (CRA) is a rare condition associated with vascular proliferation of the skin and is a manifestation of underlying localized and/or systemic comorbidities.^1^ CRA encompasses a spectrum of reactive vascular proliferations, including diffuse dermal angiomatosis, acroangiodermatitis, reactive intralymphatic histiocytosis and reactive angioendotheliomatosis. This entity is often missed during diagnosis and is often treated as cellulitis, erythema nodosum, vasculitis or contact dermatitis.^2^ CRA is difficult for clinicians to diagnose because of the rarity of the disease and its nonspecific presentation. Here we report a case report of CRA as a reactive manifestation of AML. Oftentimes, CRA is a manifestation of an underlying condition such as haematological malignancy, monoclonal gammopathy, leucocytoclastic vasculitis, hepatitis, nephritis, endocarditis or rheumatoid arthritis.^3^ The aetiopathogenesis of CRA is linked to chronic tissue imperfusion secondary to vascular occlusion, which, in turn, leads to the proliferation of vascular endothelial growth factors. In AML, a prothrombotic state is created secondary to leucostasis with the subsequent development of hypoxia-induced angiogenesis and hence the causation of CRA.^4^ The clinical feature of CRA, which is often an erythematous papulovesicular lesion with ulceration and/or necrosis, can be missed by many practising ­physicians, and patients with CRA are often mistreated for infective aetiologies, vasculopathy or contact dermatitis. The possible close clinical and histopathological mimics of this masquerading entity are Kaposi sarcoma (with typical slit-like vessel on histopathology), pyogenic granuloma (lobular capillary proliferation with plump endothelial cells) and bacillary angiomatosis (lobular ­capillary proliferation and bacterial proliferation seen on Warthin–Starry stain). This interesting case presented here reiterates the need for a vigilant clinical acumen to identify a rare but ­potentially fatal condition and the underlying associations that warrant prompt diagnosis and treatment. With proper diagnosis, early treatment can be instituted thereby preventing complications that can be debilitating and morbid if left untreated.

## Data Availability

All data in this study are included in the published article.
